# Oro-Functional Conditions in a 6-to-14-Year-Old School Children Population in Rome: An Epidemiological Study

**DOI:** 10.3390/children12030305

**Published:** 2025-02-27

**Authors:** Giuseppina Laganà, Roberta Lione, Arianna Malara, Silvia Fanelli, Francesco Fabi, Paola Cozza

**Affiliations:** 1 Department of Life Sciences, Health and Healthcare Professions, Link Campus University, Via del Casale di San Pio V, 44, 00165 Rome, Italy; g.lagana@unilink.it; 2 Department of Health Sciences, Unicamillus—Saint Camillus International University of Health Sciences, Via di Sant’Alessandro, 8, 00131 Rome, Italy; roberta.lione@unicamillus.org (R.L.); paola.cozza@unicamillus.org (P.C.); 3 Department of Systems Medicine, University of Rome Tor Vergata, Viale Oxford 81, 00133 Rome, Italy; silviafanelli7@gmail.com; 4 National Institute of Statistics (ISTAT), Central Office for Demographic Statistics and Population Census, Piazza Guglielmo Marconi, 24, 00146 Rome, Italy; francesco.fabi@istat.it

**Keywords:** epidemiological evaluation, oral conditions, dental caries, malocclusions, growing subjects

## Abstract

Background: The aim of the present cross-sectional study was to assess oral and functional conditions, the prevalence of malocclusions, and oral habits in a population of schoolchildren in Rome (Italy). Methods: The study sample included n. 1033 subjects, between 6 and 14 years of age, attending public schools in Rome. Oral health condition, occlusal relationship, and functional analysis were charted for all subjects. The rate of prevalence for the dental health element was calculated. To evaluate the relationship between the variables examined, Pearson’s Chi-square test was used to assess the significative findings of this association. Results: More than half of the students had a low level of oral hygiene (654 subjects). Class I malocclusion was the most common occlusal condition (573 subjects). Oral habits were present in most of the subjects and more than one habit was observed. Conclusions: The results of the current investigation highlight the necessity of improving public health programs for orthodontic prevention, and future screenings need to be planned to organize resources in Rome.

## 1. Introduction

Oral health is crucial to overall health and quality of life [1]. In fact, oral health promotion has played an essential role in the promotion of overall health [2] ever since the oral health and general health interrelationship was confirmed [3].

Oral health issues persist in many countries throughout the world, especially among underprivileged groups in both developed and developing countries. The significant role of socio-behavioral and environmental factors in oral disease and health is supported by many epidemiological investigations. About a quarter of five-to-six-year-old children are affected by tooth decay, and the percentage rises above 90% in some low- and middle-income countries, suggesting that dental caries is a persistent public health issue [4].

The global burden of oral conditions rose from 1990 to 2010, striking 3.9 billion people [5].

Dental caries is still a major oral health problem in most industrialized countries, affecting 60 ± 90% of schoolchildren and most adults [6]; moreover, dental care is the highest unsatisfied healthcare need [5]. The World Health Organization (WHO) estimates that dental caries in six-year-old children in European countries ranges from 20% to 90% [7]. 

Because oral health education and prevention programs for all family members, both children and parents, at all socio-economic levels are the only way to prevent dental caries [7], dentists and oral healthcare professionals give priority to oral health promotion [7], including in terms of preventing the development of malocclusions that may impair major stomatognathic functions.

Recently, the demand for orthodontic treatment has grown in the majority of countries. Epidemiological surveys are crucial to obtain comprehensive data on the prevalence of malocclusions and the social need for orthodontic treatment. This information may be used to create public health plans for dental and orthodontic prevention and screening and for organizing resources in this field [5,6,8].

Controversy still surrounds the question of the role of bad habits in the etiopathogenesis of malocclusions. Poor oral habits may interfere not only with the position of teeth but also with the normal skeletal growth pattern. Several studies have demonstrated that various environmental factors lead to malocclusion, such as eating habits and particularly the current tendency to consume soft textured foods with the reduction in masticatory forces, non-nutritive sucking, pacifier and finger sucking, and early weaning. Pacifiers, baby bottles, and mainly finger sucking often cause protrusion of the upper incisors and the premaxilla, atypical swallowing, anterior open bite, and posterior cross-bite [9].

Perrotta et al., in a study conducted in Campania (Italy) in 2018, investigated the prevalence of dental malocclusion, oral parafunctions, and TMD pain in Italian schoolchildren aged 9 to 11 years old. The study results showed that malocclusion and TMD pain are frequent findings among Italian schoolchildren and that some occlusal factors (cross-bite and open bite) and high frequency of self-reported oral parafunctions might be associated with TMD pain [10]. Another survey by Campus et al. (2007) describes the oral health status of 12-year-old Italian children according to sex, area of residence, and geographic distribution. Overall, 43.1% of the children showed dental caries, 40.5% of the male group, and 45.6% of the female group. This percentage was higher in Southern Italy than in Northern Italy [11].

Therefore, the aim of the present epidemiological study was to assess the oral conditions and the prevalence of malocclusions and to report the prevalence of oral habits in a large sample of schoolchildren in Rome (Italy). The last papers on this population in the city of Rome are dated, so this study aims to assess the actual situation in a sample of growing patients in mixed dentition.

The null hypothesis is that the prevention methods currently in place are successful.

## 2. Materials and Methods

### 2.1. Study Population

The study sample included subjects between 6 and 14 years of age attending public primary and middle schools in Rome (Italy) in mixed dentition to assess dental exchange and any alterations.

The total sample consisted of n. 1033 children (542 males and 491 females) belonging to three different educational institutions: I.C. Belforte del Chienti, I.C. Poppea Sabina, and I.C. N.M. Nicolai. The sample was collected over three different school years (2021/2022, 2022/23, and 2023/24). The schools examined were randomly chosen to reflect the appropriate distribution of socio-economic conditions in the city of Rome during the different school years. The students were selected on a voluntary basis and their personal data were collected by following an anonymous process. Children with active orthodontic treatment or cleft lip and cleft palate were not included in the elaboration of data phase.

For each participant, informed consent was requested and provided by the parents/guardians. The sample size was estimated under the assumption of a 50% prevalence ratio for any characteristic. This implies a priori unknown knowledge about the items. The confidence interval for estimating the prevalence ratio was assumed to be 95%.

This study was guided by the principles established by the World Medical Assembly in the 2008 Declaration of Helsinki on Medical Protocols and Ethics and received approval from the Ethic Committee of the Saint Camillus International University of Health Sciences (protocol number: E01015-2024).

### 2.2. Clinical Examination

The intraoral assessment was performed by five trained examiners. A pilot study on n. 50 subjects was previously performed to guarantee the accuracy of diagnosis and to standardize the methods; no statistically significant differences were found (*p* > 0.05) between the five examiners (Pearson’s Chi-square test).

Students from each class were randomly scheduled and screened by the five examiners. A research assistant monitored the examiners during the oral testing procedures. The students were examined in the medical room of the school on a chair placed close to the windows to take advantage of the natural light coming into the room. The collection phase of the clinical study was conducted in the springtime to ensure optimal climatic and lighting conditions. The examination lasted about 30 min for each subject, and the oral and occlusal conditions were recorded using latex gloves and calipers.

At the end of the medical examination, each examiner gave the child an information sheet to be given to the parents to inform them about their child's needs.

**Figure 1:**
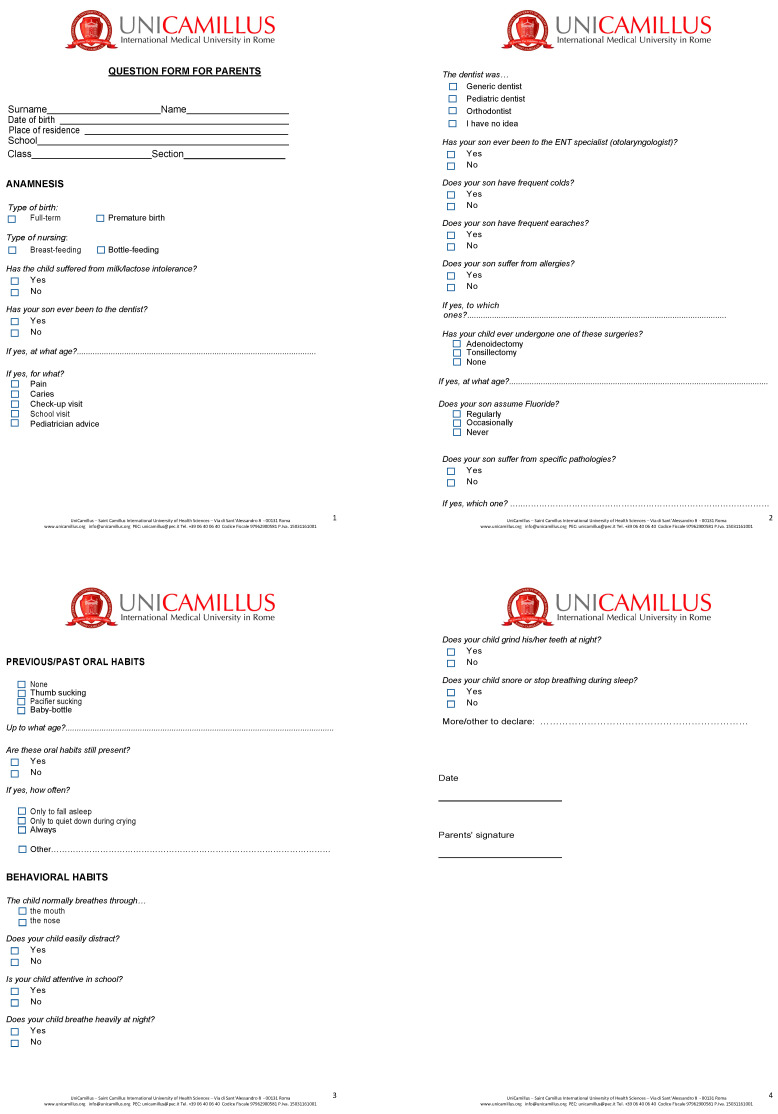
Questionnaire for the parents/guardians.

**Figure 2:**
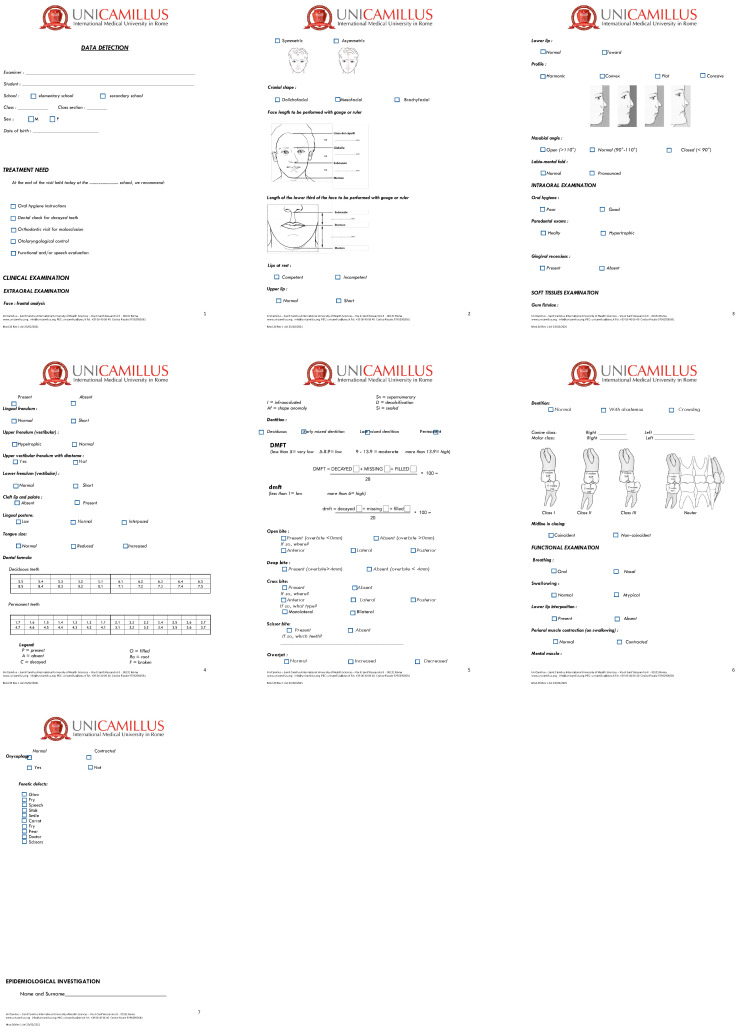
Dental chart.

The survey employed a specially prepared “guided” survey form to be followed during the visit. The form was divided into two parts as follows:

- A questionnaire, distributed in advance to the parents and/or guardians of the children involved, with open-ended questions regarding remote history and current oral and habitual behaviors (Figure 1);
- A medical record filled out by the practitioners in charge of disease detection during the visit, consisting of forty closed-ended items and divided into five parts (extra-oral clinical examination, intraoral clinical examination, soft tissue analysis, dental formula, functional clinical examination of swallowing, breathing, and phonetics) (Figure 2).

### 2.3. Statistical Analysis

Data were gathered using Microsoft Excel (version 16.61.1) and processed with the Statistical Package for the Social Sciences Windows, version 15.0 (SPSS, Chicago, IL, USA). Qualitative data were analyzed using Pearson’s Chi-square test to establish if the distributions among age, gender, and other variables were statistically different. The *p*-value for statistical significance was set at 0.05, so any value less than *p* < 0.05 was interpreted as statistically significant.

The variables considered were as follows: gender, age, oral hygiene (poor/good and plaque index), caries, (deciduous/permanent elements: dmft/DMFT), decalcifications (presence/absence of white spots and enamel demineralization areas [12]), molar class (evaluated according to Angle’s classification), dentition (normal/with diastema/crowded), vertical malocclusion (deep-bite/open-bite), transversal malocclusion (unilateral cross-bite, bilateral cross-bite, anterior cross-bite, and scissor-bite), and bad habits (finger/pacifier sucking, prolonged bottle use, bruxism, oral breathing, and onychophagy). In addition, the treatment need of each student was analyzed; in particular, the professional oral hygiene needs, dental examination (caries removal, restoration, and extraction), orthodontic treatment, ENT examination (oral breathing), and speech therapy evaluation were assessed (presence/absence of phonatory problems or atypical swallowing).

To evaluate the association between the variables examined, Pearson’s Chi-square test was used for the significative findings of this association.

## 3. Results

A total of 1033 students, aged 6 to 14 years old, were examined over the three school years. The final studied sample consisted of 542 males (52%) and 491 females (48%). Table 1 shows the composition of the sample according to age and gender collected over the three different years.

Table 2 highlights the oral hygiene status in the studied population; more than half of the students had a low level of oral hygiene.

Despite poor oral hygiene, more than 50% of the examined students showed little evidence of dental caries, as reported in Table 3.

Table 4 underlines the distribution of the sample, based on the prevalence of malocclusions. The results suggest that number of the subjects with class I malocclusion was 573 (55%), the number of subjects with class II malocclusion was 421 (41%), and 39 (4%) subjects had class III malocclusion.

Oral habits (Table 5) were present in most of the examined subjects; more than one habit was present in the same subject. Pacifier habit was the oral habit with the highest prevalence (347 subjects, 34%), followed by baby bottle habit (216 subjects, 21%) and onychophagy (180 subjects, 17%).

**Figure 3:**
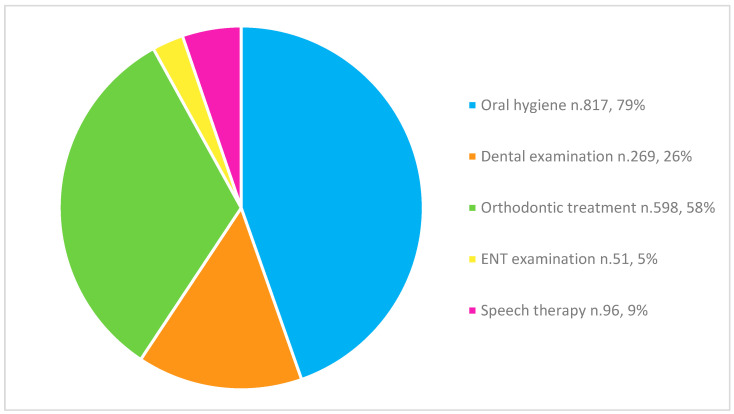
Treatment needs.

Regarding the treatment needs of every subject, the following situation was observed (Figure 3): Of the total number of subjects, 817 subjects needed a professional oral hygiene session (79%), 269 subjects needed a dental examination (26%), 598 subjects needed orthodontic treatment (58%), 51 subjects needed an otorhinolaryngological visit (5%), and 96 subjects needed speech therapy (9%). More than one specific treatment need was present in the same subject.

To evaluate the relationship between the variables examined, Pearson’s Chi-square test was used to analyze the significant findings of this association. In all three years, the following associations were found to be statistically significant (Table 6): poor oral hygiene with caries in primary and permanent teeth, dental caries in primary teeth with oral breathing, dental caries in primary teeth with pacifier sucking, dental caries in permanent teeth and dental crowding, and class III malocclusion with oral breathing.

## 4. Discussion

The current study is the first epidemiological investigation conducted on the population in the city of Rome in the last twenty years; the main goal was to obtain a true picture of the oral conditions in a group of students, aged 6 to 14 years, living in Rome. The study sample was screened to evaluate the oral conditions, the prevalence of malocclusions, and oral habits. Moreover, the statistically significant associations among all analyzed parameters were investigated.

The analysis of the results showed that most of the examined sample had poor oral hygiene (648 subjects, 63%) and were caries-free (699 subjects, 68%). In terms of malocclusions, the most frequent was class I molar (573 subjects, 55%), unilateral cross-bite (95 subjects, 9%), while most of the individuals presented normal values of overjet (570 subjects, 55%) and overbite (551 subjects, 53%). The questionnaires given to the parents were subsequently returned to the in-charge operators. Thanks to the excellent collaboration of families and from the collected data, it was possible to find the presence of interesting findings on bad habits in the examined sample; the most frequent was pacifier sucking (347 subjects, 34%).

The study sample analysis showed a moderate awareness of overall oral health, with most children showing decay-free teeth and there being a low percentage of children with caries in deciduous and permanent teeth, even if these conditions are still far from the WHO goal for 2020 (90% of the population to be caries-free) [13,14]. Specifically, the number of individuals who were caries-free was n. 116 in the 2021/2022 school year, n. 260 in the 2022/2023 school year, and n. 323 in the 2023/2024 school year. This is probably due to the fact that the water in the city of Rome contains a good amount of fluoride, which, over the years, has greatly improved the caries prevention situation in school-age children [15]. Regarding other studies in the literature, in 2021, Maldupa et al. compared the prevalence of caries in children at the age of 12 in some European countries. In Germany, 78% of children were caries-free, in Spain, 60% were caries-free, in France, 31% were caries-free, and in the UK and Latvia, 20% were caries-free [16].

In terms of oral health, though, the 2016 Global Burden of Disease Study assumed that oral diseases globally impact at least 3.58 billion people, and decay of permanent teeth was the most common of all conditions evaluated. Globally, it was assessed that 2.4 billion people have caries of permanent teeth, and 486 million children experience caries of primary teeth [14]. Dental caries is the greatest chronic disease in children, and dental care is the highest unsatisfied health need [4].

Oral health promotion has an important role in the promotion of overall health as the inter-relationship between oral and general health has been proven [17], for instance, by evidence of a significant statistical correlation between periodontitis and diabetes [4].

In the current study, dental caries in primary teeth were more frequent than on permanent teeth. In the context of oral health, primary teeth are at times considered by parents to be “practice teeth” [18] or that caries or problems in deciduous teeth can be ignored, but this is incorrect. While the deciduous teeth are present in the mouth for a brief period, they are significant for many reasons, such as helping the eruption of permanent teeth and speech development [19]. If the primary teeth are lost prematurely, such as due to decay, other primary or permanent teeth are likely to shift, or unerupted permanent teeth are likely to erupt in an incorrect position [18], resulting in crowded teeth that can be difficult to clean, possibly resulting in caries [13].

In this regard, caries is still a leading health issue in populations around the world, regardless of the success of fluoride toothpaste in decreasing its global incidence [19].

Moreover, a statistically significant association was found between caries in permanent teeth and dental crowding. Although some epidemiological studies have shown a positive association between the prevalence of dental caries and crowding (Gábris et al., 2006) [19,20,21], others have failed to establish any significant relationship (Stahl and Grabowski, 2004) [22]. Other surveys have found some associations among crowding, caries, and periodontal disease. This reinforces the point that crowded teeth render oral hygiene more difficult and might subsequently predispose the teeth to the development of dental caries, particularly when associated with poor cleaning habits [22], and this confirmed the study results. 

Another statistically significant association was found between caries in primary teeth and pacifier sucking and between primary teeth caries and oral breathing.

Early childhood caries (ECC) is a type of dental caries manifested on children’s teeth and is one of the most common dental problems during this time [23]; it is known that ECC can result in pain, infection, and interference during chewing and increase the risk of new dental caries in primary and/or permanent teeth and, sometimes, worse effects on the eruption of permanent teeth. [24].

Furthermore, environmental factors like malnutrition, genetic susceptibility, poor health status, certain eating habits, fluoride and/or vitamin D deficiency, excessive sugar consumption, and prolonged bottle-feeding or other factors (age, gender, and the continent of residence of children) are powerful factors in causing tooth decay [25]. It is noted that appropriate chewing of food plays a key role during the digestive process and for proper nutrition; teeth missing through caries can inhibit this. Connections have been established between malnutrition/poor diet and early childhood caries [26].

About dental caries and pacifier sucking, different studies have investigated if the use of a pacifier, either long term or short term and sweetened or not, is a risk factor for the development of ECC. The findings do not prove a strong or consistent relationship between pacifier use and ECC [27].

Regarding oral breathing, it is among the most widespread bad oral habits in children. It is often due to the obstruction of the upper airway, which prevents air from entering completely or partially through the oral cavity. Furthermore, as noted in the literature, oral breathing can negatively influence oral health with an increased risk of caries and periodontal diseases [28].

With regard to malocclusion, instead, knowledge of world epidemiological data helps in establishing and targeting the priorities with respect to the need for treatment, and the resources necessary to provide treatment in terms of labor capacity, skills, agility, and materials to be employed [29]. The analysis regarding the type of malocclusion showed a high presence of class I malocclusion (573 subjects, 55%), followed by class II malocclusion (421 subjects, 41%) and, lastly, class III malocclusion (39 subjects, 4%), according to the literature [30,31].

A study by Cenzato et al. demonstrated that class I malocclusion was the most frequent, in a range between 34.9% and 93.6%. The average prevalence of class II malocclusion was 20.2%. Class III malocclusion was the least frequent in all classes with an average frequency of 7.2% [26].

Moreover, the study showed a statistically significant association between class III malocclusion and oral breathing. According to Rakosi and Schilli, oral breathing could play a role in the etiopathogenesis of some forms of class III malocclusion. Mouth-breathing children have constantly open jaws and a low tongue posture with excessive mandibular growth, with the constant separation of the mandibular condyle from the fossa possibly being a growth stimulus [9].

The study of the incidence of bad habits showed the presence of different sucking noises reported by parents; the most common were pacifier and baby bottle sucking. Parents’ awareness, maximum cooperation, and positive feedback in terms of oral and occlusal health during visits facilitate greater community awareness of bad habits. This highlights how communication with families is fundamental, and this is also one of the main objectives of this work.

At the end of the medical examination, each examiner gave the child an information sheet to be delivered to the parents to inform them about their child’s needs. Most of the children were advised to proceed with further specialist visits: a total of 817 children (79%) were referred for oral hygiene and hygiene instruction sessions. A total of 269 subjects (26%) needed a dental check, 598 (58%) were referred to a specialist orthodontist, and 51 (5%) and 96 children (9%) needed an ENT and speech therapy specialist evaluation, respectively.

This action can be considered of maximum value for two reasons: direct dialogue with the families, promoting the prevention rules and behaviors at home, and, what is more, it is considered the third mission of UniCamillus in the social territory.

Lastly, it is necessary to consider some limitations of the present study. The results, which do not include the radiographic diagnosis in evaluating children with dental caries, lead to the remarkable underestimation of caries occurrence, especially with respect to the severity of caries and the interproximal sites. In addition, the students visited were preliminarily informed to obtain parental consent for the visit; therefore, the data on the level of oral hygiene are probably overestimated. It is possible that parents and children themselves are sensitized to the topic, demonstrating greater motivation and application of oral hygiene control. Furthermore, this is short-term work; at present, long-term monitoring of the participants has not been planned.

## 5. Conclusions

The results of the current investigation highlight the necessity of improving public health programs for orthodontic prevention and screening to organize and improve the resources in this urban area in Rome, especially because of the underestimation of caries due to the inability to use X-ray surveys.

School dental screening plays an important role in involving the school structure to motivate teachers and parents, who represent the main models for the children to be inspired.

An educational approach together with motivational awareness and individual reinforcement means achieving positive results by stimulating correct habits in children.

## Figures and Tables

**Table 1:** Composition of the sample by age and gender (n = 1033).

Age (Year)	Total Sample			Composition Sample by Age	Composition Sample by Gender	
	M n	F n	M + F n	M + F %	Males %	Females %
**School year 2021–2022**						
6	20	18	38	16.9	52.6	47.4
7	20	12	32	13.7	62.5	37.5
8	23	27	50	22.1	46	54
9	20	17	37	16.4	54	46
10	5	11	16	7	31	69
11	3	2	5	2.2	60	40
12	14	6	20	8.8	70	30
13	12	14	26	11.5	46	54
14	1	2	3	1.4	33	67
**Total**	**118**	**109**	**227**	**100**		
**School year 2022–2023**						
6	11	10	21	5.7	52.3	47.7
7	14	32	46	12.7	30.4	69.5
8	26	13	39	10.7	66.6	33.4
9	22	24	46	12.7	47.8	52.2
10	7	15	22	6.2	31.8	68.2
11	33	3	36	9.8	92	8
12	47	28	75	20.6	62.6	37.4
13	31	49	80	21.6	38	62
14	n.a.	n.a.	n.a.	n.a.	n.a.	n.a.
**Total**	**191**	**174**	**365**	**100**		
**School year 2023–2024**						
6	21	17	38	8.7	55	45
7	37	31	68	15.7	54	46
8	25	28	53	12.5	47	53
9	39	32	71	16.3	55	45
10	33	38	71	16.3	46	54
11	26	25	51	9.5	51	49
12	29	23	52	12.2	56	44
13	16	11	27	6.4	59	41
14	7	3	10	2.4	70	30
**Total**	**233**	**208**	**441**	**100**		

n.a.—not available.

**Table 2:** Prevalence of good/bad oral hygiene in the total sample (n = 1033).

School Year	2021–2022		2022–2023		2023–2024	
Oral Hygiene	n	(%)	n	(%)	n	(%)
Poor	127	55.9	219	60	308	69.9
Good	100	44.1	146	40	133	30.1

**Table 3:** Prevalence of dental caries in the total sample (n = 1033).

School Year	2021–2022		2022–2023		2023–2024	
Dental Caries	n	(%)	n	(%)	n	(%)
Primary teeth	78	34.4	61	16.7	79	17.9
Permanent teeth	33	14.5	44	12	39	8.8
Caries-Free	116	51.1	260	71.3	323	73.3

**Table 4:** Prevalence of malocclusions in the total sample (n = 1033).

School Year	2021—2022		2022—2023		2023—2024	
Malocclusions	n	%	n	%	n	%
Class I	154	67.8	168	46	251	57
Class II	66	29.1	187	51.2	168	38
Class III	7	3.1	10	2.8	22	5
Unilateral Cross-bite	17	7.5	32	8.8	46	10.4
Bilateral Cross-bite	8	3.5	16	4.4	6	1.4
Anterior Cross-bite	7	3.1	6	1.7	16	3.6
Scissor-bite	6	2.6	11	3	6	1.4
Normal Overjet	130	57.3	168	46	270	61
Increased Overjet	79	34.8	175	49	140	32
Reduced Overjet	18	7.9	21	5	31	7
Normal Overbite	125	55	256	70	280	63.5
Increased Overbite	85	37.5	95	26	130	29.5
Reduced Overbite	17	7.5	14	4	31	7

**Table 5:** Prevalence and distribution of oral habits in the total sample (n = 1033).

School Year	2021–2022		2022–2023		2023–2024	
Oral habits	n.	%	n.	%	n.	%
Finger	9	3.9	15	4.1	29	6.6
Pacifier	100	44	112	30.7	135	30.6
Baby bottle	63	27.7	62	17	91	20.6
Bruxism	53	23.3	26	7.1	72	16.3
Oral breathing	54	23.7	30	8.2	47	10.6
Onychophagy	6	2.60	50	13.7	124	28

**Table 6:** Association among the variables by means of Pearson’s Chi-square test.

Poor oral hygiene	Caries in primary teeth	0.002 *
Poor oral hygiene	Caries in permanent teeth	0.01 *
Poor oral hygiene	Dental crowding	0.001 *
Caries in primary teeth	Oral breathing	0.001 *
Caries in primary teeth	Pacifier sucking	0.001 *
Caries in permanent teeth	Dental crowding	0.01 *
Class III malocclusion	Oral breathing	0.002 *

* = significant interaction.

## Data Availability

Data are available from the corresponding author upon request. The data are not publicly available due to privacy reasons.
